# Surveillance of antimicrobial resistance at a tertiary hospital in Tanzania

**DOI:** 10.1186/1471-2458-4-45

**Published:** 2004-10-11

**Authors:** Bjørn Blomberg, Davis SM Mwakagile, Willy K Urassa, Samwel Y Maselle, Marcellina Mashurano, Asbjørn Digranes, Stig Harthug, Nina Langeland

**Affiliations:** 1 Centre for International Health, University of Bergen, N-5021 Bergen, Norway; 2 Institute of Medicine, University of Bergen, N-5021 Bergen, Norway; 3 Department of Microbiology and Immunology, Muhimbili University College of Health Sciences, Dar es Salaam, Tanzania; 4 Department of Microbiology and Immunology, the Gade Institute, Haukeland Hospital, N-5021 Bergen, Norway; 5 Department of Medicine, Haukeland University Hospital, N-5021 Bergen, Norway

## Abstract

**Background:**

Antimicrobial resistance is particularly harmful to infectious disease management in low-income countries since expensive second-line drugs are not readily available. The objective of this study was to implement and evaluate a computerized system for surveillance of antimicrobial resistance at a tertiary hospital in Tanzania.

**Methods:**

A computerized surveillance system for antimicrobial susceptibility (WHONET) was implemented at the national referral hospital in Tanzania in 1998. The antimicrobial susceptibilities of all clinical bacterial isolates received during an 18 months' period were recorded and analyzed.

**Results:**

The surveillance system was successfully implemented at the hospital. This activity increased the focus on antimicrobial resistance issues and on laboratory quality assurance issues. The study identified specific nosocomial problems in the hospital and led to the initiation of other prospective studies on prevalence and antimicrobial susceptibility of bacterial infections. Furthermore, the study provided useful data on antimicrobial patterns in bacterial isolates from the hospital. Gram-negative bacteria displayed high rates of resistance to common inexpensive antibiotics such as ampicillin, tetracycline and trimethoprim-sulfamethoxazole, leaving fluoroquinolones as the only reliable oral drugs against common Gram-negative bacilli. Gentamicin and third generation cephalosporins remain useful for parenteral therapy.

**Conclusion:**

The surveillance system is a low-cost tool to generate valuable information on antimicrobial resistance, which can be used to prepare locally applicable recommendations on antimicrobial use. The system pinpoints relevant nosocomial problems and can be used to efficiently plan further research. The surveillance system also functions as a quality assurance tool, bringing attention to methodological issues in identification and susceptibility testing.

## Background

Exaggerated and irrational use of drugs, availability of antibiotics without prescription, the use of pharmaceuticals of doubtful quality and the HIV epidemic may all contribute to the current worldwide surge in antimicrobial drug resistance. Emerging resistance to antimicrobial drugs increases morbidity and mortality by hampering the provision of effective chemotherapy, and makes treatment more costly [1-3]. The surge in antimicrobial resistance seen in many low-income countries is potentially disastrous because of the lack of resources for purchasing expensive second-line drugs [4].

It is widely held that surveillance of antimicrobial susceptibility is fundamental to combat the emergence of resistance [5]. Surveillance must be global since resistant bacteria can be transferred between countries, but it must also be local, since countries have very different resistance patterns and different treatment practices [6]. The primary task of a surveillance system is to provide locally applicable data to guide empiric therapy. Furthermore, surveillance may help assessing the magnitude of the resistance problem locally, nationally and internationally, monitoring changes in resistance rates and detecting the emergence and spread of new resistance traits. A well-functioning surveillance system is also necessary to measure the impact of any interventions. Surveillance systems also functions as a quality assurance tool and may help improving the quality of the susceptibility testing.

This paper describes the experience with the implementation of a computerized surveillance system for antimicrobial drug susceptibility at Tanzania's major referral hospital, and its use to analyze the susceptibility patterns of 7621 consecutively recorded clinical bacterial isolates.

## Methods

### Setting

The study was performed at Muhimbili National Hospital (MNH), Dar es Salaam, Tanzania. With more than 1000 beds, MNH is the largest hospital in the country and serves as a national referral and university teaching hospital, as well as a primary and referral hospital for a population of approximately 3.6 million in the Dar es Salaam area. The Department of Microbiology and Immunology at MNH examines specimens from inpatients and outpatients at MNH, and from a number of nearby hospitals. Bacteriological cultures are performed on more than 23,000 specimens per year.

### The surveillance system

A free-of-charge software for the surveillance of antimicrobial resistance (WHONET, World Health Organization, Geneva, Switzerland) [7] was implemented at MNH in 1998. Currently a total of 880 microbiology laboratories in 76 countries use this software, however, among these are only 41 laboratories in four countries on the African continent (data from 2002, personal communication from John Stelling, author of the WHONET software). The software has three main parts, a laboratory configuration file which can be used to customize it to the particular laboratory, an interface for data entry and a part for analysis and reporting of resistance data. At our hospital, all bacterial isolates of clinical significance from specimens received during the period July 1^st ^1998 to December 31^st ^1999 were recorded and analyzed. The specimens examined included urine, pus/secretions (swabs from skin, surgical and traumatic wounds, burns, umbilical cords, throat, nose, eye and ear discharge and genital swabs), blood, cerebrospinal fluid, other body fluids, stools and other specimens. Mycobacteria and anaerobic bacteria were not included in the study. Apart from the WHONET software, we used Stata 8.0 for Macintosh (Stata Corporation, College Station, Texas, USA) to evaluate differences of proportions by Fisher's exact test (2-tailed, cut-off point for statistical significance at p-value of 0.05).

### Laboratory methods

The specimens were cultured and the bacterial isolates identified using standard microbiological methods as described in Mackie & McCartney Practical Medical Microbiology [8]. Susceptibility testing was performed by Stokes' method [9] on Iso-Sensitest (Oxoid Limited, Basingstoke, UK) agar plates. This method, developed by Dr Joan Stokes half a century ago, was designed to monitor for both disc and agar quality in that both the clinical isolate and a control strain were tested on every plate. The clinical isolate is swabbed onto the middle of the agar plate and the control strain at the periphery. The antibiotic disk is placed precisely at the interface between the surface areas inoculated with the clinical isolate and the control strain. After overnight incubation, the relative size of the inhibition zones of the clinical isolate and the control strains are compared. The test results are classified as susceptible (S), intermediate (I) or resistant (R) by evaluation of the difference between the inhibition zones of the clinical isolate and the control strain. The control strains used in our lab are *S. aureus *NCTC 6571, *E. coli *NCTC 10418 or *Pseudomonas aeruginosa *NCTC 10662. The isolates showing intermediate resistance were few and were grouped together with sensitive isolates for the purpose of data analysis. Either methicillin or oxacillin disks were used to test for methicillin-resistance in *S. aureus*, the results being considered equivalent and interchangeable in the data analysis. ß-Lactamase testing was not routinely performed. The susceptibility of pneumococci to penicillin was examined by the use of penicillin 2 μg disks. Commercially produced antibiotic disks, mostly obtained from Oxoid Limited, were used, however, in some instances, antibiotic disks, prepared locally were used due to financial constraints. The Department of Microbiology and Immunology participates in an external quality assessment program in bacteriology led by the World Health Organization-collaborating centre, the National Institute for Communicable Diseases (NICD), Johannesburg, South Africa. The Department of Microbiology and Immunology at our hospital receives bacterial strains from NICD, performs species identification and antimicrobial susceptibility testing, and report the results back to NICD.

### Evaluation of the surveillance system

We evaluated the strengths and shortcomings of the surveillance system in our setting, particularly in terms of how well it performed in its main application areas, providing locally applicable data to guide empiric therapy, monitoring antimicrobial susceptibility trends, detecting the emergence and spread of new resistance traits and as a tool for quality assurance. We also assessed the cost-implications of implementing the surveillance program in our setting. We considered direct costs, such as the purchase of equipment, and indirect costs, such as those related to the running of the laboratory, including human resources. We also comment on the benefits of the surveillance system related to both direct patient care and long-term implications of containing antimicrobial resistance.

## Results

### Bacterial isolates

A total of 7617 bacterial isolates were registered during the study period, of which 67.4% (n = 5134) were Gram-negative and 32.6% (n = 2483) Gram-positive. Table 1 shows the most frequently encountered bacteria, overall and from various specimen types. The majority of the isolates were obtained from pus (44.3%), urine (43.5%) and blood cultures (10.1%). Cerebrospinal fluid accounted for 0.4% of the isolates. Among the 2034 blood cultures, 15.9% (n = 323) yielded growth of a total of 326 pathogenic bacterial isolates and 447 Coagulase-negative staphylococci (CoNS) as shown in Table 1. CoNS are potential pathogens and are increasingly considered as a cause of blood-stream infections. However, in many cases they are merely contaminants, i.e. bacterial isolates present on the skin surface, which are introduced in the blood specimen and grow in the blood culture, but do not produce disease in the patient. For CoNS isolates to be considered a probable pathogen, it is commonly required that they are recovered from two separate blood cultures. Since multiple blood cultures were not routinely taken from the same patient in the hospital, the susceptibilities of these isolates were not evaluated further. CoNS and various other Gram-positive probable contaminants, mostly *Bacillus *spp. were recovered from 22.0% (n = 447) and 6.9% (n = 141) of the blood cultures, respectively. Furthermore, five *Candida *spp. isolates and one *Cryptococcus neoformans *were recovered. Among the 49 *Salmonella *isolates, two were identified as *S*. Typhi, 16 as *S*. Typhimurium, 16 as *S*. Paratyphi B and one each as *S*. Paratyphi C, *S*. Enteritidis and *S*. Arizonae. Twelve Salmonella isolates were not serotyped. Among the 41gonococcal isolates, 28 (68.3%) were from genital swabs. Eleven (26.8%) gonococcal isolates were obtained from the neonatal ward, out of which 4 were specified as from eye discharge.

**Table 1:** Frequency of pathogenic^a ^bacterial isolates from different specimen types at Muhimbili National Hospital, Tanzania

**Organism**	**Blood (%)**	**Spinal fluid (%)**	**Urine (%)**	**Pus^b^(%)**	**Other (%)**	**Overall (%)**
**Gram-negative isolates**						
*E. coli*	27 (3.5)	0 (0.0)	1466 (44.2)	417 (12.3)	26 (21.8)	1936 (25.4)
*Klebsiella *spp.	91 (11.8)	8 (23.5)	1036 (31.3)	603 (17.9)	33 (27.7)	1771 (23.3)
*Pseudomonas *spp.	10 (1.3)	2 (5.9)	52 (1.6)	531 (15.7)	9 (7.6)	604 (7.9)
*Proteus *spp.	7 (0.9)	0 (0.0)	121 (3.7)	249 (7.4)	3 (2.5)	380 (5.0)
*Enterobacter *spp.	4 (0.5)	0 (0.0)	97 (2.9)	1 (0.0)	0 (0.0)	102 (1.3)
*Salmonella *spp.	37 (4.8)	2 (5.9)	6 (0.2)	0 (0.0)	4 (3.4)	49 (0.6)
*N. gonorrhoeae*	0 (0.0)	0 (0.0)	0 (0.0)	41 (1.2)	0 (0.0)	41 (0.5)
*Haemophilus *spp.	1 (0.1)	5 (14.7)	0 (0.0)	0 (0.0)	0 (0.0)	6 (0.1)
Other GNR	32 (4.1)	7 (20.6)	12 (0.4)	184 (5.4)	10 (8.4)	245 (3.2)
Subtotal, Gram-negative isolates	209 (27.0)	24 (70.6)	2790 (84.2)	2026 (60.0)	85 (71.4)	5134 (67.4)
**Gram-positive isolates**						
*Staphylococcus aureus*	72 (9.3)	1 (2.9)	362 (10.9)	1120 (33.2)	12 (10.1)	1567 (20.6)
*Streptococcus pyogenes*	1 (0.1)	0 (0.0)	0 (0.0)	160 (4.7)	2 (1.7)	163 (2.1)
Other streptococci^c^	39 (5.0)	3 (8.8)	52 (1.6)	58 (1.7)	13 (10.9)	165 (2.2)
Enterococci	3 (0.4)	0 (0.0)	64 (1.9)	3 (0.1)	1 (0.8)	71 (0.9)
*S. pneumoniae*	2 (0.3)	6 (17.6)	0 (0.0)	11 (0.3)	6 (5.0)	25 (0.3)
CoNS^a^	447 (57.8)	...	45 (1.4)	...	...	492 (6.5)
Subtotal, Gram-positive isolates	564 (73.0)	10 (29.4)	523 (15.8)	1352 (40.0)	34 (28.6)	2483 (32.6)
Total	773 (100.0)	34 (100.0)	3313 (100.0)	3378 (100.0)	119 (100.0)	7617 (100.0)

GNR, Gram-negative rod-shaped bacteria, not further identified; CoNS, coagulase-negative staphylococci; "...", not applicable.^a ^CoNS from blood and urine specimens are reported as possible pathogens, although many may be contaminants. CoNS from other specimen types are considered contaminants and not reported. ^b ^Pus includes swabs from skin, surgical and traumatic wounds, burns, umbilical cords, throat, nose, eye and ear discharge and genital swabs. ^c ^Streptococci other than *S. pyogenes *and *S. pneumoniae*, and streptococci not identified below genus level.

Specimens from inpatients and outpatients contributed to 53.2% and 31.9% of the isolates, respectively. A further 6.0% were obtained from specimens from other hospitals in Dar es Salaam, while 8.8% were obtained from other or unknown locations. Among the isolates from inpatients, 36.5% were obtained from the Department of Pediatrics, 28.4% from the neonatal section and 8.1% from the other pediatric wards. The other isolates came from the Departments of Surgery (22.4%), Internal Medicine (16.6%), Obstetrics and Gynecology (9.8%), the Intensive Care Unit (4.9%) and other locations (9,8%). For 4900 isolates, the age or the estimated age group of the patient was known. Of these, 23.6% (n = 1155) were from neonates (≤ 1 month old), 6.8% (n = 335) from children aged one month to seven years, and 69.6% (n = 3410) from adults or children older than 8 years.

### Antimicrobial susceptibility

Tables 2 and Table 3 show the antimicrobial susceptibility patterns of the most frequently isolated Gram-negative and Gram-positive bacteria, respectively. There were no clear-cut differences in the antimicrobial susceptibilities among the various serotypes of *Salmonella *isolates (data not shown). The majority of *Pseudomonas aeruginosa *isolates was susceptibility-tested to gentamicin only, to which 4.3% (15/350) were resistant. Among the isolates of *Neisseria gonorrhoeae*, 70.0% were resistant to penicillin, 45.2% to tetracycline, 59.3% to trimethoprim-sulfamethoxazole, 5.9% to erythromycin and none was resistant to spectinomycin, fluoroquinolones or amoxicillin-clavulanate (data not shown).

**Table 2:** Percentage of Gram-negative bacterial isolates resistant to antimicrobial agents (number of tested isolates in brackets)

**Drug**	***E. coli***	***Klebsiella* spp.**	***Proteus* spp.**	***Enterobacter *spp.**	***Salmonella* spp.**	**GNR**
Ampicillin	80% (1761)	85% (1572)	60% (331)	72% (86)	70% (46)	56% (204)
Amoxicillin- clavulanate	28% (1292)	32% (1153)	17% (247)	32% (78)	52% (23)	31% (124)
Ceftazidime	5% (788)	6% (605)	2% (95)	10% (51)	0% (8)	14% (35)
Tetracycline	77% (1223)	66% (1016)	77% (211)	72% (54)	42% (12)	45% (153)
Gentamicin	8% (1634)	14% (1538)	7% (343)	15% (91)	9% (23)	8% (217)
Trimethoprim- sulfamethoxazole	76% (1313)	69% (1174)	57% (224)	70% (56)	73% (44)	51% (172)
Sulfonamides	84% (174)	84% (231)	74% (46)	100% (14)	95% (22)	62% (34)
Nitrofurantoin	32% (929)	53% (652)	72% (71)	48% (48)	...	...
Chloramphenicol	45% (250)	51% (372)	55% (132)	...	20% (41)	57% (138)
Fluoroquinolones	13% (432)	6% (343)	3% (65)	6% (32)	0% (20)	15% (40)
Nalidixic acid	28% (509)	16% (334)	18% (22)	31% (16)	...	...

GNR, Gram negative rod-shaped bacteria, not further identified; "...", not tested.

**Table 3:** Percentage of Gram-positive bacterial isolates resistant to antimicrobial agents (number of tested isolates in brackets)

**Drug**	***S. aureus***	**CoNS**	**Enterococci**	***S. pneumoniae***	***S. pyogenes***	**Other strept.^a^**
Penicillin	97% (1521)	93% (42)	67% (9)	4% (23)	0% (163)	23% (98)
Ampicillin	...	...	6% (66)	...	...	13% (83)
Methicillin/ cloxacillin	2% (1556)	21% (47)	...	...	...	...
Tetracycline	49% (1042)	90% (39)	76% (51)	8% (13)	47% (131)	61% (90)
Erythromycin	29% (1543)	69% (48)	26% (65)	6% (18)	7% (161)	26% (156)

CoNS, Coagulase-negative staphylococci; "...", not tested. ^a ^Streptococci other than *S. pyogenes *and *S. pneumoniae*, and streptococci not identified below genus level.

Comparison of resistance patterns of isolates obtained from inpatients and outpatients at MNH did not show large differences. However, ampicillin resistance was more frequent in urinary isolates of *E. coli *from inpatients than in those from outpatients as shown in Table 4. Likewise, urinary isolates of *Klebsiella *spp. from inpatients were more frequently resistant to gentamicin and trimethoprim-sulfamethoxazole than isolates from outpatients.

**Table 4:** Percentage of urinary *E. coli *and *Klebsiella *spp. isolates from inpatients and outpatients resistant to antimicrobial agents

	***E. coli***			***Klebsiella *spp.**		
**Drug**	**Inpatients**	**Outpatients**	**P^a^**	**Inpatients**	**Outpatients**	**P^a^**

Ampicillin	87.2	82.7	0.036^a^	92.2	91.1	0.624
Amoxicillin- clavulanate	31.4	28.3	0.344	37.7	33.9	0.327
Ceftazidime	4.9	5.6	0.731	7.6	6.0	0.577
Tetracycline	83.1	81.7	0.648	82.0	75.2	0.053
Gentamicin	8.6	7.7	0.572	14.9	5.4	<0.001^a^
Trimethoprim- sulfamethoxazole	86.0	81.3	0.067	82.7	74.2	0.012^a^
Sulfonamides	92.1	87.8	0.510	95.2	100.0	0.553
Nitrofurantoin	33.7	33.1	0.881	52.1	58.0	0.157
Fluoroquinolones	17.8	12.7	0.217	7.2	6.7	1.000
Nalidixic acid	29.0	28.2	0.913	14.0	18.8	0.334

^a ^P < 0.05 (Fisher's exact test, 2-tailed) indicates statistical significance of the differences in resistance rates.

Comparison of resistance patterns in isolates blood cultures with those from other specimen types showed apparent great differences for some drugs, however, in most cases the number of blood culture isolates were few and did not show statistically significant differences. However, as shown in Table 5, blood culture isolates of *Klebsiella *spp. were indeed more frequently resistant to gentamicin than those from other specimen types. A significantly greater proportion of blood culture isolates of *S. aureus *were resistant to tetracycline than among those from other specimen types, whereas for penicillin the isolates from blood cultures were resistant in a lower proportion than the others.

**Table 5:** Percentage of bacterial isolates from different specimen types resistant to antimicrobial agents

	***E. coli***			***Klebsiella *spp.**			***S. aureus***		
**Drug**	**Blood**	**Other**	**P^a^**	**Blood**	**Other**	**P^a^**	**Blood**	**Other**	**P^a^**

Penicillin	...	...	...	...	...	...	91.5	96.9	0.028^a^
Ampicillin	84.0	79.4	0.803	84.3	85.3	0.759	...	...	...
Amoxicillin- clavulanate	40.0	27.8	0.383	29.8	31.9	0.873	...	...	...
Methicillin	...	...	...	...	...	...	1.4	2.2	1.000
Ceftazidime	0.0	5.3	1.000	5.7	6.0	1.000	...	...	...
Tetracycline	54.5	77.3	0.139	66.7	66.4	1.000	84.6	48.3	<0.001^a^
Erythromycin	...	...	...	...	...	...	21.1	29.0	0.179
Gentamicin	13.0	7.7	0.416	41.3	12.3	<0.001^a^	...	...	...
Trimethoprim- sulfamethoxazole	72.0	76.3	0.636	63.0	69.1	0.297	...	...	...
Sulfonamides	83.3	84.0	1.000	86.8	83.4	0.809	...	...	...
Chloramphenicol	58.3	43.8	0.199	57.9	49.3	0.200	...	...	...
Fluoroquinolones	40.0	13.1	0.136	0.0	6.5	0.381	...	...	...

"...", not applicable. ^a ^P < 0.05 (Fisher's exact test, 2-tailed) indicates statistical significance of the differences in resistance rates.

### Evaluation of the surveillance system

A great number of bacterial isolates were recorded in the system. All age groups and both inpatients and outpatients were represented in the study. More than a third of the isolates were from outpatient populations from the Dar es Salaam area, however we cannot exclude the possibility of a selection bias in favour of patients with infections caused by resistant organisms, since many patients get treatment at primary health facilities before reaching MNH. We do not know how well the rural population is represented in this material, but we assume that the outpatients in the study are mostly from the Dar es Salaam area. Ten percent of the isolates represented systemic infections, i.e. isolates from blood cultures and spinal fluid. The susceptibility test results were recorded as interpreted values (i.e. "R" (resistance), "I" (Intermediate) or "S" (susceptible)) and not as inhibition zone diameters. In this study, no molecular techniques were available for the detection of resistance genotypes and evaluation of genetic relatedness of bacterial isolates.

The direct cost of implementing the surveillance system was limited to the purchase of a computer at approximately 1000 Euro. However, less expensive second-hand computers would be sufficient. The software was downloaded free of charge from the WHO website. The indirect costs of running this surveillance program are related to human sources for operating the software, including data entry and analysis, and the costs of the susceptibility testing activities. It is difficult to separate these indirect costs from the costs of running the daily laboratory activities. In our setting, a laboratory technologist from the department took on the task of operating the software in addition to her regular duties. In our experience, for a hospital of our size, it is recommendable to allocate approximately 50% of a laboratory technologist position to operating the surveillance software. In our setting, this would translate into a monthly cost of approximately 100 Euro for the department. The surveillance system is dependent on susceptibility testing of acceptable quality. The susceptibility testing incurs costs related to human resources and the purchase of laboratory reagents including antimicrobial disks and agar media. Implementing a surveillance system may increase these costs by focusing on the importance of quality reagents. However, since the susceptibility testing activities are an integral activity of the department, which would have been performed regardless of the surveillance system, we choose not to attribute their costs to the surveillance system in this context. The benefits of a surveillance system are difficult to quantify, but are of potentially great magnitude. Foremost, surveillance data may improve empiric therapy for infections and thus save lives and reduce suffering. It may reduce treatment costs by enabling the use of the least expensive effective drugs. Additionally, surveillance systems may contribute to containing or reducing antimicrobial resistance, which in the long term perspective may have great benefits in reducing morbidity and mortality, and diminish the need for expensive second-line antimicrobial agents.

The strengths and weaknesses are elaborated on in the Discussion part.

## Discussion

### Resistance patterns and implications for therapy

Experience from the World Health Organization's External quality assurance system for antimicrobial susceptibility testing has shown that disk diffusion testing is suitable for routine surveillance [10]. However, disk diffusion is not optimal for testing of certain important resistance traits, such as penicillin-resistance in pneumococci. The lack of international standardization of methods and interpretive criteria causes concern, but there are indications that routine susceptibility testing data are suitable for surveillance even if obtained with different methods [11].

Consistent with observations from a number of other countries in the region [12-15] and elsewhere [16], Gram-negative bacilli displayed high rates of resistance to common inexpensive antibiotics. Reasonably priced antibiotics such as ampicillin, tetracycline, trimethoprim-sulfamethoxazole and sulfonamides are now of limited benefit in the treatment of infections caused by important Gram-negative bacteria such as *E. coli*, *Klebsiella *spp., *Proteus *spp. and *Salmonella*. Chloramphenicol may fail to cure as much as a quarter of infections caused by *Salmonella *and half or more of infections caused by *E. coli*, *Klebsiella *spp. and *Proteus *spp. Fluoroquinolones appear to be the only reliable drugs for oral treatment of infections caused by common Gram-negative bacilli, whereas gentamicin and third-generation cephalosporins remain useful for parenteral therapy.

The study showed a very low prevalence of methicillin-resistant *S. aureus*, consistent with previous data from the same hospital [17,18]. While the results should be interpreted with some caution since confirmatory nucleic acid based techniques were not available, the data support the current use of isoxazolyl penicillins, such as cloxacillin for the treatment of staphylococcal infections at the hospital. There were few isolates of enterococci compared to studies from high-income countries [19]. It is reassuring that the current study showed a low rate of ampicillin-resistant enterococci, indicating that nosocomial infections caused by these micro-organisms is a minor problem compared with many high-income countries. Low consumption of broad-spectrum antibiotics such as third-generation cephalosporins, fluoroquinolones, imipenem and vancomycin may explain this finding [19-21]. While other countries in the region have been affected by penicillin-resistant pneumococci [22,23], the current study indicates that pneumococcal disease in Dar es Salaam can safely be treated with penicillin or erythromycin. However, the results should be interpreted with some caution since the number of isolates tested was small. More than a quarter of the gonococcal isolates (11/41) were obtained from the neonatal ward, and most or all of these isolates probably represent gonococcal conjunctivitis. Amoxicillin-clavulanate, spectinomycin, fluoroquinolones and erythromycin appear to be good alternatives for the treatment of gonococcal infections. An apparent increase in resistance to trimethoprim-sulfamethoxazole (from 18% to 59%) is noted since the study by Mbwana [24] from 1993 to 1995, however, this may be due to the use of different methodology for susceptibility testing.

### Applicability of data to guide treatment of serious infections

Recommendations for antibiotic treatment of serious bacterial infections such as bloodstream infections and meningitis should preferably be based on knowledge of the prevalence and antimicrobial susceptibility patterns of pathogens isolated from blood and spinal fluid. While a fair number of bacterial isolates were tested in the current study, the number of blood culture isolates was limited (n = 329, excluding the CoNS isolates). As shown in Table 5, there appears to be differences in resistance between isolates obtained from blood cultures and those from other specimen types, but these are difficult to assess because of the low number of blood culture isolates. Thus, the data from the current surveillance should be interpreted with caution with regards to the treatment of serious infections. The CoNS isolates obtained from blood were recorded in the WHONET database, since they may represent clinically important infections such as bacteremia in patients with compromised immunity, patients with indwelling intravascular devices and the newborn [25]. The study showed that a high proportion (21.9%) of blood culture bottles yielded CoNS isolates. However, the conventional way to distinguish pathogenic isolates of CoNS from contaminants, by requiring growth of a similar CoNS isolate in a separate blood culture, could rarely be used, since follow-up cultures were seldom available. Consequently, the susceptibilities of these isolates were not evaluated further.

### Relevance of data for outpatient and rural populations

It is important to specify for which population the surveillance data are valid. At our hospital, specimens from both inpatients and outpatients were examined. The hospital is to a great extent used as a primary hospital for the population in the Dar es Salaam area. However, among the cases coming to the hospital, there may be a degree of selection of patients with infections caused by resistant microbes, since many patients rely on health centers and pharmacies to cure simple ailments, and only come to the hospital when primary treatment fails. The study found that a few resistance traits, such as ampicillin resistance in *E. coli *and gentamicin and trimethoprim-sulfamethoxazole resistance in *Klebsiella *spp. were more frequent in urinary isolates from inpatients than from outpatients. Apart from that, there were no dramatic differences between isolates from inpatients and outpatients. The data from the study should be representative for both the hospital setting and to some degree the population in Dar es Salaam. However, the majority of the population of Tanzania lives in rural areas, where resistance patterns may be substantially different. Thus one should be cautious to extrapolate the results of the current study to be valid for populations in the countryside.

### Ability to monitor trends of antimicrobial susceptibility

Certain trends in antimicrobial susceptibility could be identified by comparison with data from other studies. While resistance to ampicillin, tetracycline and sulfonamides in Gram-negative bacteria was frequent already in the seventies [26,27], it is worrying that resistance to trimethoprim-sulfamethoxazole, chloramphenicol, nitrofurantoin, nalidixic acid and amoxicillin-clavulanate appear to have increased compared to previous studies [27,28]. The extensive use of chloramphenicol for the treatment of presumed cases of typhoid fever and the use of trimethoprim-sulfamethoxazole for the ambulatory treatment of chest infections, malaria and other infections, may have contributed to the high prevalence of resistance to these two drugs. Although still low, it is of concern that the rate of gentamicin-resistance in *E. coli *has increased from zero in 1978–79 [27] to 2% in 1995 [28] and 8% in the current study. In neighboring Kenya, the rate of gentamicin-resistance in *E. coli *has increased from 2% in the late seventies [29] to 20% and above in recent studies [12].

Resistance to gentamicin is common in Gram-negative bacteria with extended-spectrum beta-lactamases (ESBL), sometimes in as much as 96% of isolates [30]. Such an association cannot be investigated in the current study, since less than half of the isolates of *E. coli *and *Klebsiella *spp. were tested for susceptibility to third-generation cephalosporins and other methods for detection of ESBL (double disk synergy test, Etest, PCR) were not available.

Also in *P. aeruginosa *the rate of gentamicin-resistance has increased, from zero in the seventies [27] to 4% in the current study. Resistance to penicillin and erythromycin was common among *S. aureus *isolates in this study. However, the rate of tetracycline resistance (49%) was lower than reported from the same hospital in 1979 (57%) and 1982 (74%) [17]. In the late seventies, tetracycline was used in great quantities in Tanzania to prevent and treat cholera; as much as 1788 kilograms of the drug were used during a period of only 5 months [31]. Due to the rapid emergence of tetracycline-resistant *Vibrio cholerae*, the use of the drug was subsequently greatly reduced, and this may have contributed to a concurrent decline in the rate of tetracycline-resistance in an unrelated species such as *S. aureus*.

For meaningful comparison of data from different studies, whether from the same or different laboratories, the same method of susceptibility testing should preferably be employed. In our laboratory, the same method has been used for a number of years. The WHONET software features a number of sophisticated ways to analyze susceptibility information based on the measurements of inhibition zone diameters. Recording the diameter of the inhibition zones in disk diffusion testing is generally recommended [32], and may increase the accuracy of results and enable the detection of gradual shifts in antibiotic susceptibility over time. It also makes the data independent of the current breakpoints. With the WHONET software, data can easily and rapidly be re-analyzed with reference to new breakpoints. However, the Stokes' method for susceptibility testing [9], which is used in our laboratory, is based on visual interpretation of the difference in inhibition zones between the clinical isolate and the control strain. The interpretation is recorded as interpreted values, i.e. either susceptible "S", intermediate susceptible "I" or resistant "R". The WHONET software also accepts susceptibility data to be entered and analyzed as "interpreted values", i.e. "S", "I" and "R". The use of such interpreted values enables most of the analysis features of WHONET, but not all. Foremost, analyzing data based on zone diameters (or MIC values) is superior for the early detection of subtle shifts in antimicrobial resistance over time, which may alert clinicians about emerging resistance trends at an early stage. However, one asset of the Stokes' method, particularly under tropical conditions, is that unsuspected poor antibiotic disk quality will be discovered quickly since a control strain is tested on every plate.

Furthermore, variations over time in the battery of antibiotics tested makes comparison of data less useful. Laboratories in low-income countries are sometimes vulnerable to this because of unreliable supplies of antibiotic discs.

### Ability to detect emerging resistance traits

Disk diffusion testing may give indications of emerging resistance traits such as methicillin-resistance in *S. aureus *and ESBL in Gram-negative bacteria. The current surveillance indicated that methicillin-resistance is rare in *S. aureus *at the hospital. Ideally this should be confirmed with PCR-based methods to detect the *mec*A gene. Likewise, the disk diffusion testing showed the presence of resistance to ceftazidime in Gram-negative isolates, albeit at a low rate, which calls for further investigation with regard to the possible presence of ESBL. Our laboratory did not employ molecular methods for detection of resistance genes on a routine basis, but a recent study showed low prevalence of methicillin-resistant *S. aureus *(MRSA) [18]. Resistance surveillance should be coupled with awareness of signs of various resistance traits and, preferably, the possibility of using molecular methods to verify emerging resistance traits.

### Ability to detect nosocomial problems

The WHONET software is well suited to analyze antibiograms in order to detect suspicious nosocomial outbreaks. These functions too are dependant on the use of a consistent battery of test drugs, and also works better when results are entered as actual values for MIC or zone diameters, as opposed to the interpreted value ("S", "I" or "R"). In our hospital, comparison of resistance rates did not show dramatic variation between isolates from inpatients and outpatients. The exception was a trend for more frequent gentamicin-resistance in inpatient isolates of Gram-negative bacteria, particularly *Klebsiella *spp., which may suggest possible nosocomial spread. The analysis of antibiograms did not produce convincing evidence of clonal patterns spread of bacterial isolates, possibly partly due to the variations in the battery of antibiotics tested. Molecular methods for the evaluation of the genetic relatedness of bacteria were not available in this study.

### Suitability for international comparison of resistance data

In 2002 a total of 880 laboratories in 76 countries across the world used the software, including 41 laboratories in 4 African countries. The WHONET system has been implemented at MNH since 1998. Unfortunately, there is no international consensus on a recommended method for antimicrobial susceptibility testing. Worldwide at least twelve different *in vitro *methods are followed, and only in Europe the number is at least ten [5]. Furthermore, there are ongoing changes in the interpretive criteria for susceptibility testing [10]. In addition to this, there is an abundance of molecular methods to describe various genetic markers of resistance. *In vivo *clinical assessment is of great importance in understanding bacterial drug resistance and the gold standard for evaluating resistance in malaria parasite. The multitude of methods employed for antimicrobial susceptibility testing has to some extent hampered the meaningful sharing and comparison of resistance data among countries. Recently, much work has been done in Europe to harmonize resistance surveillance efforts across country borders [33,34]. While many laboratories record inhibition zones for disk diffusion results, interpretation is usually according to national guidelines. Thus, susceptibility patterns from different countries must be compared prudently. The lack of standardization in methods is a problem that must be addressed at an international level.

### The surveillance system as a quality assurance tool

The implementation of the surveillance system brought focus on methodological issues, including microbial identification and susceptibility. The WHONET software has built-in functions to alert the operator if isolates with unexpected resistance patterns are entered. During the surveillance exercise in our laboratory, it was discovered that four isolates of *Streptococcus pyogenes *were reported as resistant to penicillin. This was subsequently double-checked, and consulting the laboratory bench-book we found that clerical errors were the explanation for this. The use of the surveillance software enabled the easy detection, investigation and correction of such errors, and consequently may contribute to increase the attention to quality issues and generally improve the performance of the lab. The current surveillance project highlighted some methodological issues, most of which were caused by budgetary limitations, such as the occasional use of locally made antibiotic disks and limitations in the identification of organisms due to lack of reagents.

### Impetus for further research

Routine surveillance makes use of available large data sets at little additional cost and may be representative for a greater part of the population. However, often it is necessary to supplement the routine surveillance with ad hoc studies aimed at investigating particular problems. While ad hoc studies generally are more expensive to conduct, they allow for the use of more advanced and expensive laboratory methods and are better at targeting particular populations of interest. The current surveillance study identified a need for more data from bloodstream infections in order to provide reliable guidance for the treatment of serious bacterial infections. As a consequence of this, we started a study of bloodstream infections with the pediatric department at the hospital. Another laboratory-based research was started to ascertain the finding that methicillin-resistance in staphylococci is still relatively infrequent at this hospital.

### Influencing popular opinion on antimicrobial resistance issues

Resistance surveillance is a platform from which to promote focus on antimicrobial resistance issues, both within the hospital and the medical community, but also among the general population. In conjunction with the surveillance exercise, we have highlighted issues regarding antimicrobials and resistance in local newspaper letters [35], and there is ongoing work to establish a chapter of APUA (Alliance for the prudent use of antibiotics, ) in Tanzania.

### Cost considerations and human resources

The study suggests that laboratories, which perform susceptibility testing, can gain useful information on antimicrobial susceptibility with a minimal budget. As appropriate software can be obtained free-of-charge, the main cost of the surveillance system is associated with purchasing a computer. However, there are other, indirect costs, which may be attributed to the surveillance program depending on the situation of the laboratory, such as running costs for microbiologic procedures, including susceptibility testing. Particularly, it is important to ensure availability of antimicrobial discs of satisfactory quality. A susceptibility surveillance system also implies the need for some additional human resources for data entry and analysis. In our experience, it is recommendable to allocate approximately 50% of a laboratory technologist position to this task. While the WHONET program is excellent for entry, analysis and reporting of resistance data, the software is not intended to function as a complete patient management system for the laboratory. Data can be transferred from other databases into WHONET by the use of a complementary software called BacLink (also free-of-charge). However, in laboratories such as ours, where the management of patients' laboratory tests (i.e. receipt of specimens and laboratory forms, inscription in registers, return of test results, etcetera) is handled manually via register-books, the data must be punched into WHONET by hand. Since the WHONET database is not used directly for patient management, the surveillance activity tends to become less integrated in the clinical routine work than it should. Thus, although the program performs its task very well, in a long-term perspective, a surveillance system that is integrated with a patient management system might be more sustainable. It is difficult to quantify the potential benefits of a well-functioning surveillance system. However, we are fully convinced that the modest costs of the surveillance program are highly justified since the data generated may improve empiric therapy, help contain or prevent the further emergence of antimicrobial resistance, decrease the need for expensive second-line antimicrobial drugs and, ultimately, save lives and reduce suffering.

## Conclusions

It is imperative to preserve the effectiveness of common antibiotics by promoting rational use of antibiotics based on sound knowledge of local resistance patterns. In a hospital with bacteriology services, the implementation of a computerized surveillance system is a low-cost tool to make use of available resistance data. In our hospital, the resistance surveillance system has generated information on resistance patterns that is useful as guidance for empiric therapy of infections. It can help alert clinicians of trends of antimicrobial resistance, guide drug-policy decisions and facilitate rational use of drugs to prevent the further emergence of antimicrobial resistance. The surveillance system has also served as a quality assurance tool and led to increased focus on antimicrobial resistance and prudent use of drugs. There is need for more data from blood cultures for reliable guidance for the treatment of severe, systemic bacterial infections. For antibiotic policy recommendations to be applicable for the general population, more information is needed from outpatients and rural areas.

There is limited information on antimicrobial resistance trends on the African continent. Only four African countries use the WHONET system for antimicrobial resistance surveillance, although some countries may use other similar software. Recently much work has been done to establish consensus and a more standardized approach to resistance surveillance in Europe [34]. Susceptibility data based on recorded zone diameters, instead of interpreted values ("S", "I" and "R"), would make the surveillance system more effective in detecting subtle changes in antimicrobial resistance. We believe there is a need for a standardized approach to antimicrobial resistance surveillance also in the African region, as well as globally. This would facilitate liaisons and sharing of information among countries.

## Competing interests

The authors declare that they have no competing interests.

## Authors' contributions

BB was the principal investigator, participated in the planning and execution of the study, performed data entry and data analysis, and was the main responsible author. DSMM, WU, SYM and AD participated in the planning of the study and contributed to the writing process. MM contributed to designing the WHONET database, performed data entry and microbiological work, and contributed to the writing process. SH participated in the writing. NL was the project coordinator and participated in planning, data analysis and writing.

## Pre-publication history

The pre-publication history for this paper can be accessed here:


